# *SiDT1* Defines Plant Architecture Reminiscent of Green Revolution in Foxtail Millet

**DOI:** 10.1002/advs.76506

**Published:** 2026-07-14

**Authors:** Jianzhen Lv, Jinjin Cheng, Zhen Hu, Qian Lan, Yanjun Yang, Chengcheng Pei, Junhao Luo, Zhuang Li, Peiyong Xin, Jijun Yan, Jinfang Chu, Qian Qian, Zhaosheng Kong, Liang Jiang

**Affiliations:** ^1^ Hou‐Ji Laboratory in Shanxi province Shanxi Agricultural University Taiyuan China; ^2^ College of Agriculture Shanxi Agricultural University Taiyuan China; ^3^ National Center for Plant Gene Research (Beijing) Institute of Genetics and Developmental Biology, CAS Beijing China; ^4^ Yazhouwan National Laboratory Sanya China

## Abstract

Foxtail millet (*Setaria italica*) is a drought‐tolerant C_4_ cereal that grows on marginal lands and serves as a nutrient‐rich food for millions in Asia and Africa. Despite its resilience and nutritional value, the genetic basis underlying plant height variation in foxtail millet remains incompletely understood, thereby constraining the effective implementation of semi‐dwarfing strategies analogous to those that drove the Green Revolution in major cereals. This study identified *sidt1*, a semi‐dwarf, high‐tillering mutant exhibiting a compact architecture and enhanced lodging resistance. It is demonstrated that *SiDT1* encodes a GA3‐oxidase orthologous to rice *D18/XIAOWEI*. A single A‐to‐T mutation disrupted its catalytic function, reducing bioactive gibberellin biosynthesis. CRISPR‐Cas9 knockout lines recapitulated the *sidt1* phenotype, confirming *SiDT1*’s functional role. Combined transcriptomic profiling and SiD53 immunoblot analysis indicated that strigolactone‐related signaling is perturbed in *sidt1*, in agreement with its enhanced tillering phenotype. Notably, under high‐density planting conditions, *sidt1* maintained grain yield and quality while exhibiting superior lodging resistance. These findings identify SiDT1 as a key regulator of plant architecture and establish a semi‐dwarf ideotype reminiscent of the rice Green Revolution, providing a valuable genetic resource for high‐density and mechanized foxtail millet production.

## Graphical abstract

*SiDT1* encodes a GA3‐oxidase that creates a semi‐dwarf, lodging‐resistant architecture reminiscent of the rice Green Revolution. The resulting ideotype performs well under dense planting and provides a valuable genetic resource for high‐yield, mechanized foxtail millet production.

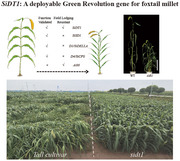

## 1 Introduction

Foxtail millet (*Setaria italica*), a cereal crop of the Poaceae family, was domesticated from its wild progenitor, green foxtail (*Setaria viridis*) [1, 2]. It occupies a unique position in global agriculture because of its adaptation to marginal, rain‐fed and semi‐arid environments, as well as its low input requirement [3, 4]. These characteristics establish foxtail millet as resilient staple for smallholder systems in parts of Asia and Africa, and an important crop for food‐security strategies under climate change scenarios [5]. In addition to its resilience, foxtail millet contributes to dietary diversity and human nutrition: its grain contains appreciable levels of protein, dietary fiber and micronutrients (e.g., iron, zinc) and a range of bioactive phytochemicals, making it attractive for functional‐food and health‐oriented markets [6]. Furthermore, owing to its small genome and expanding genomic toolkit, foxtail millet and its wild progenitor green foxtail have emerged as C4 model organisms for fundamental plant science research, thereby augmenting both its academic significance and translational potential [7, 8, 9, 10, 11].

Archaeobotanical and molecular evidence indicates that foxtail millet was domesticated in China, estimates commonly place domestication for over 11 500 years [1]. Domestication in foxtail millet, compared to its wild ancestor green foxtail, included a suite of classical syndromes such as reduced seed shattering [12], enhanced apical dominance [13], increased seed size [14], and the loss of photoperiod sensitivity [15]. This domestication process was not a singular or uniform event. Rather, a complex history of initial domestication followed by regional adaptation produced a mosaic of distinct landraces and ecotypes, each fine‐tuned to local microclimates [15]. The long cultivation history and local selection have generated substantial genetic diversity that is a resource for modern breeding. The initial haplotype map of foxtail millet was constructed utilizing 0.8 million prevalent single nucleotide polymorphisms (SNPs) through the sequencing of 916 diverse foxtail millet varieties sourced from various regions across China, in which 512 putative loci associated with 47 agronomic traits were identified by genome‐wide association studies (GWAS) [16]. Recently, a graph‐based reference genome integrating 113 reference‐grade genomes consisting of 36 wild, 41 landrace, and 36 modern cultivated *Setaria* accessions have been achieved, revealing a multitude of structural variants underlie the phenotypic adaption and domestication [17]. Besides morphological change, a handful of metabolites have been altered during foxtail millet domestication [18, 19]. GWAS have revealed genes involved in the biosynthesis pathways of flavonoids and lignin that may in turn affect millet color [19]. Notably, the PHYTOENE SYNTHASE1 (PSY1), the first committed and rate‐limiting step in carotenoid biosynthesis, is essential for the yellow grain color [19, 20].

As the domestication center of foxtail millet, China had a long foxtail millet breeding history. Throughout the era spanning the Warring States period to the Northern and Southern Dynasties (c. 475 BCE–589 CE), foxtail millet consistently held the primary position in grain production [21]. Over centuries of cultivation, ancient Chinese agriculturalists accumulated extensive expertise in foxtail millet husbandry, which was systematically summarized by Sixie Jia during the Northern Wei Dynasty (386‐534 CE). Foxtail millet declined sharply from the Ming Dynasty onward, the ongoing expansion of staple cereal rice and wheat cultivation gradually diminished its dominance [22]. For example, foxtail millet remained the principal cultivated crop in North China in 1949, with a sown area of 9.207 × 10^6 ^ha [23]. However, its cultivation area has reduced persistently, stabilizing at around 0.8 × 10^6 ^ha in recent years. Although conventional approaches such as selection from landraces, pedigree crossing, and recurrent selection have generated many locally adapted cultivars with enhanced stability, maturity and some disease resistance, their yield per unit area remains lower than that of major cereals (rice, wheat and maize). Recent advances in foxtail millet breeding have involved the adoption of hybrid breeding systems and molecular tools in selected programs to enhance the rate of genetic improvement [24]. To bridge the yield gap, strategic priorities continue to include investments in systematic trait mapping, identification of major effect loci, particularly in plant architecture.

Plant architecture, the integrated suite of traits that determine plant form (e.g., plant height, tillering, leaf angle, and inflorescence structure), is a principal determinant of harvest index and yield potential [25]. Architectural modifications that reduce lodging risk and redirect biomass toward grain permit denser planting and more intensive input use, thereby raising attainable yields. The Green Revolution (beginning in the 1960s) exemplified this relationship: the breeding of semi‐dwarf wheat and rice varieties reduced lodging and increased the proportion of biomass allocated to grain, which in turn enabled higher planting densities and substantially greater yield responses to fertilizer and irrigation [26, 27]. Between 1960 and 2000, average yields per hectare in developing countries rose markedly—by approximately 208% for wheat, 109% for rice, and 157% for maize—with particularly large gains in Southeast Asia, India, and China [28, 29]. Although lodging‐resistant foxtail millet germplasm was reported as early as 1961, the underlying genetic basis for those materials remains unavailable. In recent decades, several dwarfing loci in foxtail millet have been cloned or molecularly characterized. For example, *D1*, a homolog of rice *SLR1*, causes dwarfism via an N‐terminal deletion of the DELLA domain following insertion of a retrotransposon [30]. *D2* is a recessive dwarf gene encoding a cytochrome P450 implicated in phytohormone biosynthesis [31]. A single‐nucleotide G deletion in exon 3 of *Seita.5G404900*, which is the foxtail millet ortholog of the rice Green Revolution gene *SD1*, causes a frameshift mutation that introduces two amino acid substitutions and a premature stop codon. Consequently, the encoded protein is truncated to 342 amino acids instead of the 423 amino acids in the wild‐type form [32]. Notwithstanding these molecular advances, the agronomic performance and contribution of these cloned loci under field conditions remain largely uncharacterized. *SiDWARF4* encodes the ent‐copalyl diphosphate synthase (CPS) enzyme involved in gibberellin (GA) biosynthesis in foxtail millet [33]. Conversely, the semi‐dwarf line *Ai88*—valued by breeders for its compact architecture—was gradually adopted as a backbone parent in foxtail millet breeding programs because of its favorable field performance; however, the causal gene(s) controlling the reduced height of *Ai88* have not yet been identified [34]. Taken together, these observations highlight a gap between gene discovery and validated utility in production breeding, underscoring the need for field‐level evaluation and functional dissection of widely used dwarf germplasm [35].

In this report, we identified and characterized a semi‐dwarf, increased‐tillering mutant of foxtail millet, designated *sidt1*, which displays a compact architecture and enhanced lodging resistance. The causal locus, *SiDT1*, encodes a GA3‐oxidase orthologous to rice *D18*/*XIAOWEI* [36, 37], a single A to T transition abolishes its catalytic activity, thereby diminishing the biosynthesis of bioactive gibberellins. Notably, under high‐density planting, *sidt1* conferred lodging resistance with no yield penalty, and did not compromise grain quality. These findings reveal *SiDT1* as a central regulator of plant architecture, paralleling the semi‐dwarfism central to Green Revolution rice.

## 2 Results

### 2.1 *SiDT1* Showed Resistance to Lodging under Field Conditions

**FIGURE 1:**
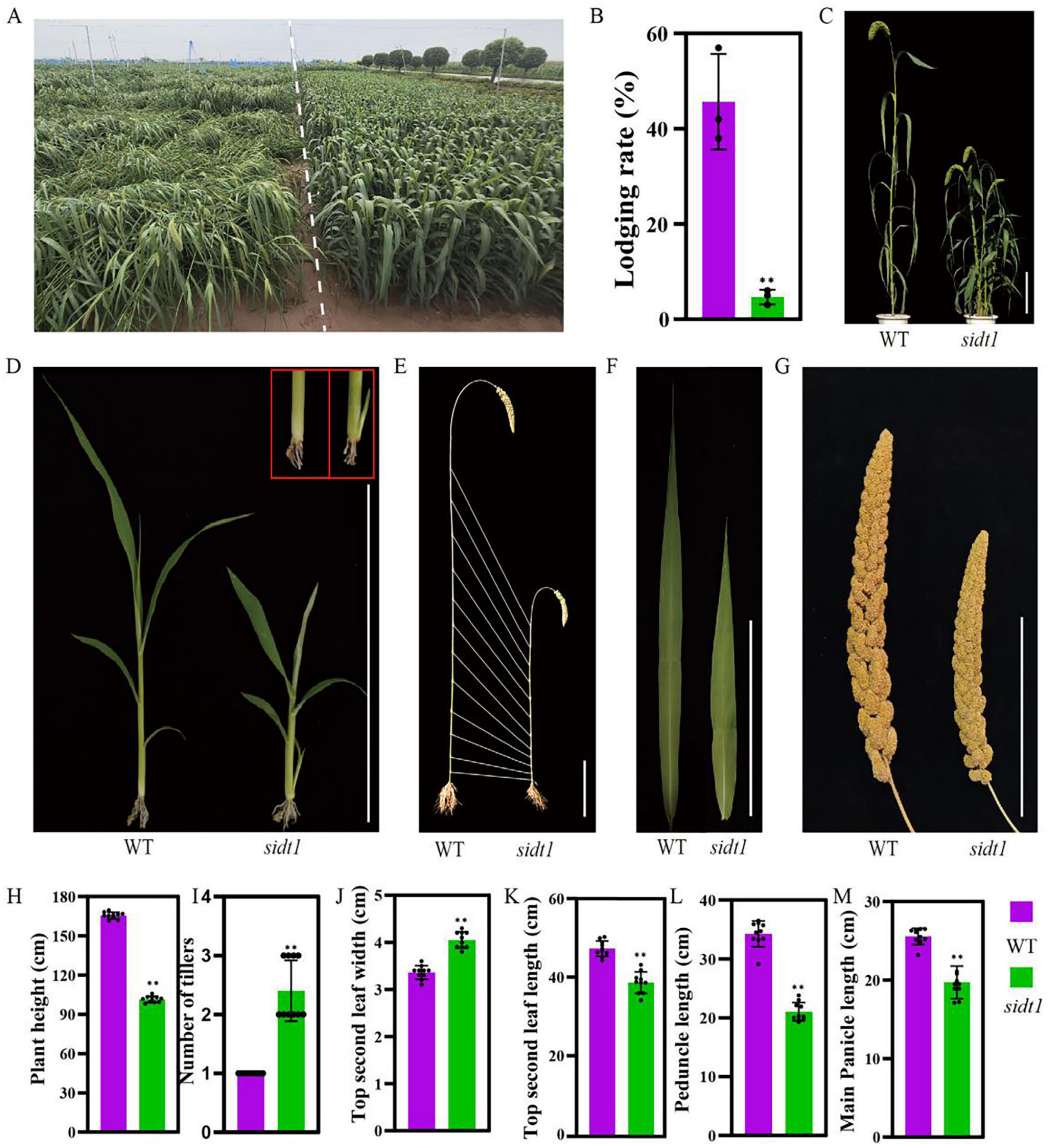
*sidt1* conferred enhanced lodging resistance under field conditions. (A) Comparison of the lodging phenotype between the high plant height cultivar (left) and the *sidt1* mutant (right). (B) Statistical analysis of lodging rate between wild‐type Jingu40 and the *sidt1* mutant, based on data from three independent field plots (*n* = 3). (C–G) Phenotypic comparison between wild‐type Jingu40 and the *sidt1* mutant showing mature plants (C), 2‐week‐old seedlings (D), internode length (E), leaf shape (F), and panicle architecture (G). From C to G, all scale bars represent 20 cm. Bar charts show quantitative differences in major agronomic and architectural traits between WT (purple) and *sidt1* (green) plants, Plant height (H), Tiller number (I),Top second leaf width (J), Top second leaf length (K), Peduncle length (L), Panicle length (M). From H to M, *n* = 10. Data are presented as mean ± standard error (SE). Student's t‐test (*, *p* < 0.05; **, *p* < 0.01).

In northern China, the filling stage of foxtail millet typically occurs in July, when heavy rains accompanied by strong winds caused severely stem lodging, particularly in tall varieties such as Jingu21, one of the most widely cultivated varieties in China (Figure S1A,B). To address this lodging susceptibility, we identified an EMS‐induced mutant, *sidt1* (***S*** *etaria* ***i*** *talica* ***d*** *warf and* ***t*** *illering* ***1***), derived from the elite cultivar Jingu40. Under field conditions, during a severe storm event, *sidt1* displayed substantially enhanced lodging resistance compared with its tall wild‐type (Figure 1A). The lodging rate of *sidt1* was reduced to ≈3%, in sharp contrast to the ≈45.6% observed in Jingu40 and other tall cultivars (Figure 1B). In addition to its lodging resistance, *sidt1* displayed a distinct plant‐architecture phenotype: average plant height was ≈100 cm for *sidt1* compared with ≈160 cm for wild‐type Jingu40 under the same field conditions, and tiller number in *sidt1* was 2.2‐fold higher than in Jingu40 (Figure 1C,D). The shortened stature of *sidt1* is attributable to reduced internode length rather than a decrease in internode number (Figure 1E). Leaf length and panicle size were reduced in *sidt1* relative to Jingu40 (Figure 1F,G). The root architecture of *sidt1* was comparable to Jingu40 under field condition (Figure S1C). Histological analysis suggested that the cell size is reduced in *sidt1* mutant culms, leading to dwarfism in the mutant plant (Figure S2). The combination of shorter stature and increased tillering confers the observed lodging resistance in *sidt1* (Figure 1H–M and Figure S1D).

### 2.2 Cloning and Characterization of *SiDT1*

**FIGURE 2:**
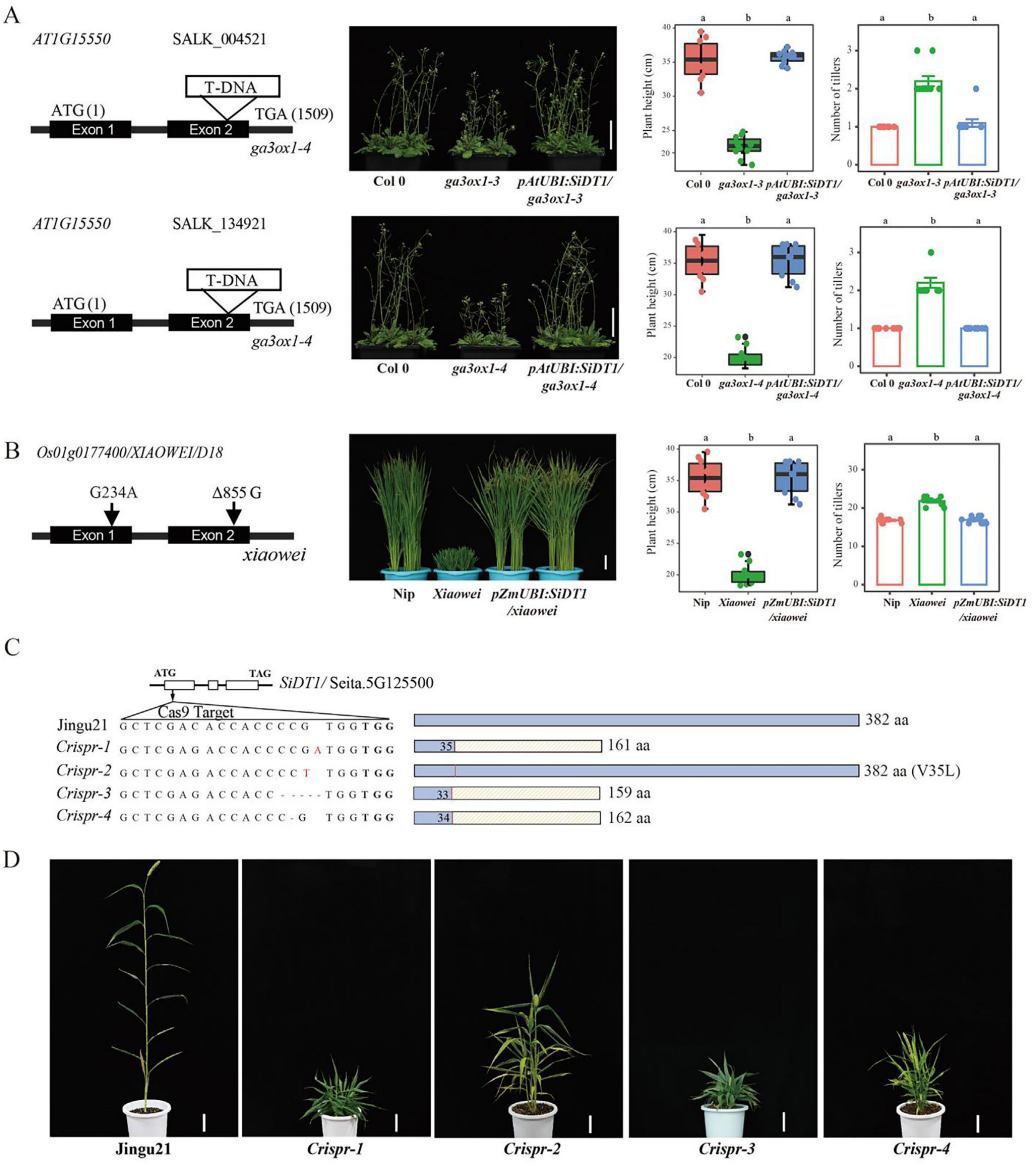
Genetics confirmation of *S iDT1*. (A) The Arabidopsis *ga3ox1* mutants were complemented by the heterologous expression of the *pAtUBI10:3F‐SiDT1*. Plant height and branch number were analyzed (*n* = 10). Representative images of plants are shown. (B) The rice *ga3ox2/d18/xiaowei* mutants were complemented by the heterologous expression of the *pZMUBI:3F‐SiDT1*. Plant height and tiller number were analyzed (*n* = 10). representative images show the phenotypic differences. (C) Design of CRISPR‐Cas9 target sites and generation of homozygous mutant lines. The grey‐blue box denotes the wild‐type amino acid sequence. The hatched box represents the frameshift mutation, which leads to a premature termination codon. (D) The phenotype of CRISPR‐Cas9 generated mutants. All scale bars represent 10 cm.

To isolate the *SiDT1* gene, we took a map‐based cloning approach. Genetic analysis of a backcross‐derived F_2_ population (*n* = 290) yielded 85 mutant individuals, a segregation pattern consistent with control by a single locus (χ^2^ = 2.872, 0.05 < *p* < 0.1) (Figure S3). *SiDT1* was initially localized to an interval of ∼2 Mb between the two molecular markers M3 and M8 on the short arm of chromosome 5. To fine‐map the *SiDT1* locus, we generated a large F_2_ mapping population derived from a cross between *sidt1* and Yugu1. The *SiDT1* locus was located between the two SSR markers M5 and M6 (Figure S4A). Combined transcriptomic analysis and Sanger sequencing identified an A‐to‐T substitution at nucleotide 838 in the third exon of *Seita.5G125500*, and this substitution results in a N to Y amino acid substitution at 280 position (Figure S4A). *Seita.5G125500* encodes a protein belonging to the 2‐oxoglutarate (2OG)‐and Fe (II)‐dependent dioxygenase (2ODD) family, catalyzing the oxidation of substrate using 2OG and molecular oxygen as co‐substrates and ferrous Fe(II) cofactors (Figure S4B). A BLASTP search revealed that *Seita.5G125500* shares 60% amino acid identity with *Seita.3G103700* in foxtail millet. SiDT1 had the greatest similarity to GA 3β‐hydroxylase sequences of enzymes that have been reported in rice, *Arabidopsis*, and other crops (Figure S4B). Phylogenetic analysis grouped SiDT1 within the GA3 OXIDASE 2 clade, distinct from the GA3 OXIDASE 1 clade (Figure S4C). *SiDT1* expression level is high in seed germination, medium in shoot and root, and low in sheaths, and leaves (Figure S5). Due to the challenges of genetic transformation in the *sidt1* mutant (in the Jingu40 background), we first performed a functional complementation assay by expressing the *SiDT1* cDNA in the corresponding *Arabidopsis* mutant. In *Arabidopsis*, gibberellin 3β‐hydroxylase is encoded by a multigene family comprising at least four members (Figure S4C). We analyzed two previously reported T‐DNA insertion alleles of *ga3ox1* (SALK_004521 and SALK_134921) [38], which exhibited semi‐dwarf phenotypes, with a ≈50% reduction in final plant height and slightly smaller rosettes compared to the WT. Constitutive expression of *SiDT1* in both *Arabidopsis* mutants fully rescued the dwarf phenotype (Figure 2A). To test functional conservation in a monocot system, we also expressed *SiDT1* constitutively in the rice *d18*/*xiaowei* mutant, which is defective in the orthologous GA3ox2 gene. Similarly, *SiDT1* expression rescued the dwarf phenotype in rice (Figure 2B). Collectively, these complementation assays demonstrate that the biological function of *SiDT1* is conserved across distantly related species, including eudicots (*Arabidopsis*) and monocots (rice). To further validate its function in its native species, we generated *SiDT1* knockout mutants in foxtail millet using the clustered regularly interspaced palindromic repeats (CRISPR)‐associated protein 9 (Cas9) technique. A single‐guide RNA (sgRNA) targeting the early coding region of *SiDT1* was designed and cloned into the pHUE411‐*SiDT1* vector. The construct was then introduced into the highly transformable foxtail millet cultivar Jingu21 via Agrobacterium‐mediated transformation. From the T_1_ generation, we identified four homozygous mutant lines. Among these, three mutant lines carried frame shift mutations (caused by single‐base‐pair indels) leading to premature termination codons, and one line contained a missense mutation resulting in a valine to leucine (V to L) substitution (Figure 2C). All frameshift mutant lines exhibited a severe dwarf phenotype compared to the wild‐type Jingu21 (Figure 2D). Notably, the V to L substitution conferred a moderate dwarf phenotype without obvious growth penalties, suggesting that this allele may be more suitable for practical breeding applications. This result confirms that disruption of *SiDT1* is sufficient to cause dwarfism in foxtail millet.

### 2.3 GAs Levels Altered in *sidt1* Because of SiDT1^N280Y^ Mutation

**FIGURE 3:**
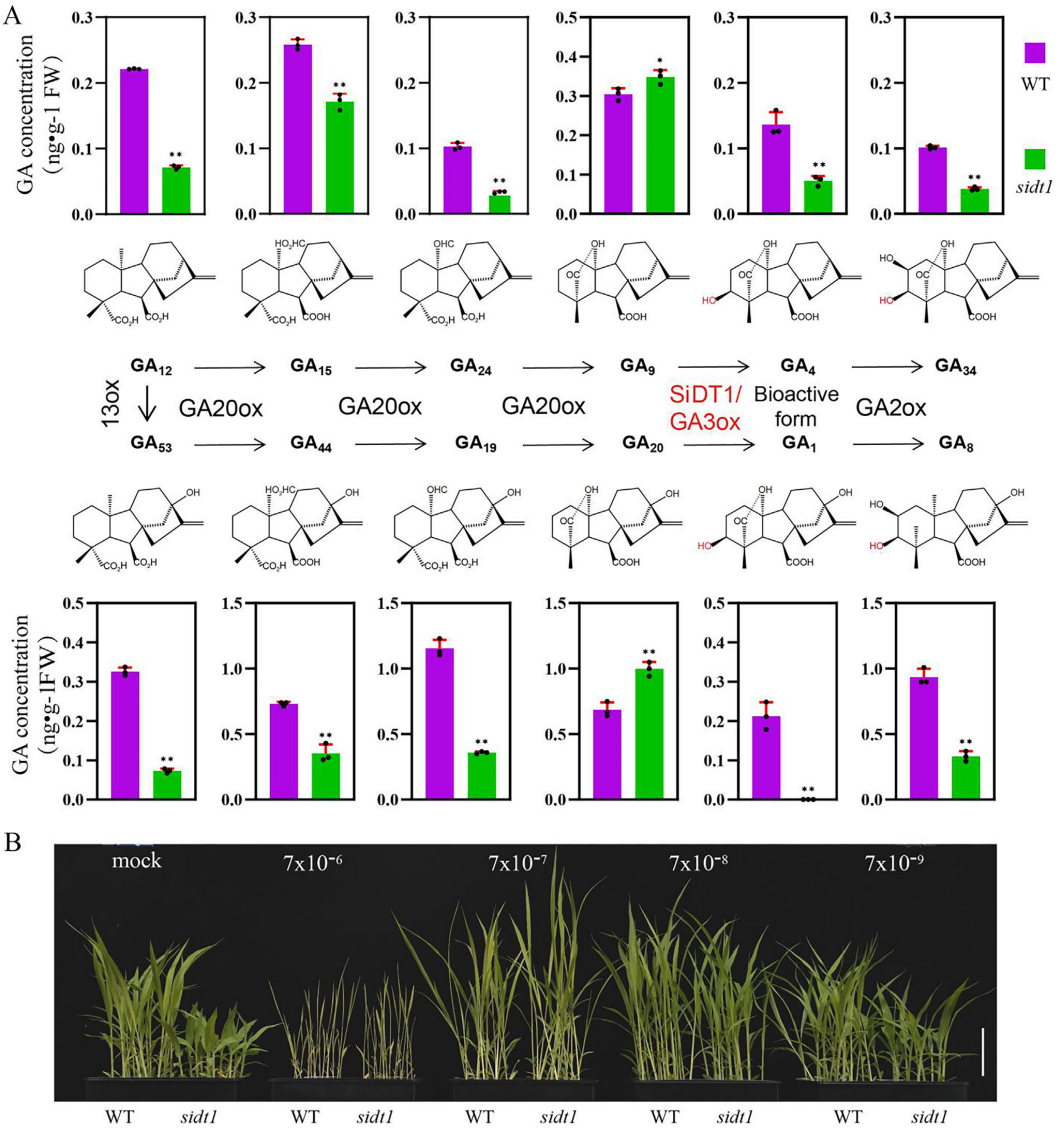
GAs levels altered in *sidt1*. (A) Simplified GA metabolic pathway in flowering plants. Key GA metabolic steps and their associated intermediates are shown. Bar plot shows the quantified levels of endogenous GAs in wild‐type (WT) and *sidt1* mutants (*n* = 3). **, *p* < 0.01. (B) Exogenous GA_3_ application rescues the growth phenotype of the *sidt1* mutant. Nine‐day‐old WT and *sidt1* seedlings were transferred to media supplemented with bioactive GA_3_ at various concentrations. Scale bars = 10 cm.

**FIGURE 4:**
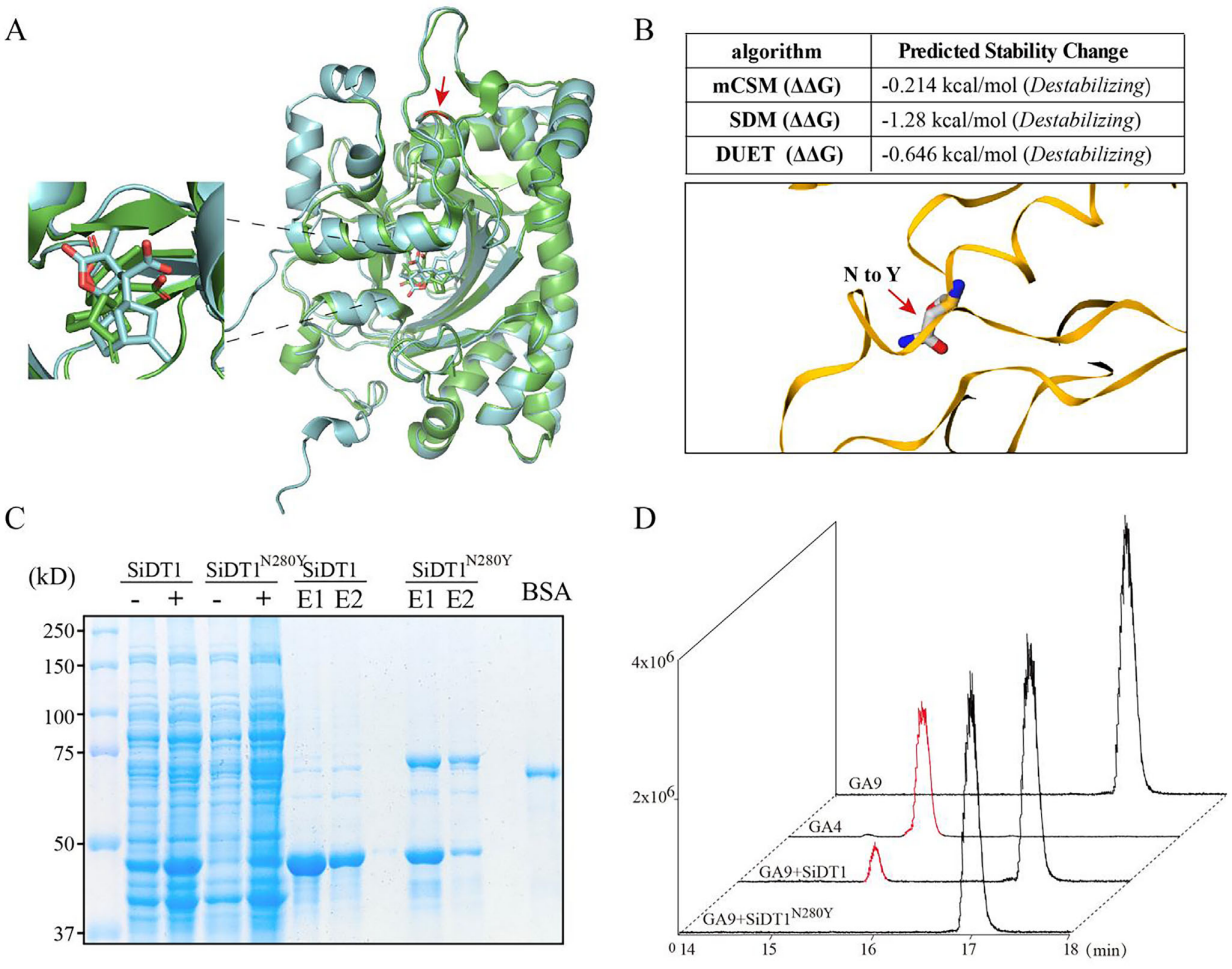
The SiDT1^N280Y^ mutation abolishes its catalytic activity. (A) Molecular docking of GA9 into the SiDT1 protein model. The protein structure of SiDT1 was predicted using AlphaFold3. The resolved crystal structure of OsGA3ox2 (green) was obtained from the Protein Data Bank (PDB). The molecular structure of gibberellin A9 (GA9) was retrieved from the PubChem database. (B) DUET prediction of structural stability changes induced by the N280Y mutation in SiDT1. Three computational tools (mCSM, SDM, and DUET) were used to predict mutation‐induced changes in protein stability (∆∆G in kcal/mol). (C) The SiDT1 protein was heterologously expressed in *E. coli*. Under same culture conditions and with equivalent bacteria, the SiDT1^N280Y^ mutant protein showed reduced abundance compared to the wild‐type. (D) Representative LC/MS chromatograms of the in vitro reaction products of GA9 catalyzed by SiDT1 and SiDT1^N280Y^.

The mutated residue in *SiDT1* is evolutionarily conserved across grass species, suggesting its critical role for protein function (Figure S4B). As GA3OXs catalyze the final step in the synthesis of bioactive gibberellins, we investigated the metabolic consequences of the mutation. We quantified endogenous GA levels in 22‐day‐old seedlings. The results showed that the bioactive GA_4_ content in *sidt1* was 0.05 ng g^−1^ FW, 60% lower than in the wild type (0.12 ng g^−1^ FW) (Figure 3A). Notably, another bioactive form, GA_1_, was nearly undetectable in the mutant (Figure 3A). Consistently, the immediate substrates of SiDT1, GA_9_ and GA_20_, accumulated in *sidt1*. Furthermore, earlier precursors in the non‐13‐hydroxylation pathway (GA_12_, GA_15_, GA_24_) that lead to GA_9_ were reduced in the mutant. These data confirm that the dysfunction of SiDT1 disrupts the GA biosynthetic pathway, leading to a severe deficiency in bioactive GAs. Similarly, precursors in the 13‐hydroxylation pathway, including GA_20_, accumulated in the *sidt1* mutant (Figure 3A). This coordinated decrease in early precursors (from GA_12_ to GA_24_ and from GA_53_ to GA_19_, respectively) is likely attributable to a negative feedback regulation of *GA20ox* transcript levels, a mechanism previously documented in rice [36, 39, 40]. These GA profiling results indicated that the dwarf phenotype of *sidt1* is caused by a deficiency in bioactive GAs. To further substantiate this, we applied GA_3_, a bioactive gibberellin, to wild‐type and *sidt1* seedlings in hydroponic culture. Exogenous GA_3_ at concentrations ranging from 7.0 × 10^−6 ^M to 7.0 × 10^−8 ^M fully rescued the dwarf phenotype of 2‐week‐old *sidt1* seedlings (Figure 3B). These results demonstrate that SiDT1 is essential for GA production in foxtail millet, and the SiDT1^N280Y^ mutation is the direct cause of the GA deficiency. To elucidate the molecular mechanism by which the N280Y mutation impairs function, we predicted the SiDT1 protein structure using AlphaFold3. The structure reveals an internal cavity that likely forms part of the gibberellin‐binding pocket (Figure 4A). Notably, residue N280Y is distant from the predicted active site, suggesting it is not directly involved in substrate binding. However, in silico analysis indicated that the N280Y substitution may reduce protein stability (Figure 4B). To test this, we expressed and purified recombinant SiDT1 and SiDT1^N280Y^ protein, respectively. We observed lower yields of the mutant protein under the same conditions, supporting that the mutation compromises stability (Figure 4C). We proceeded to compare the catalytic activities of the purified proteins in vitro. When incubated with GA_9_, the wild‐type SiDT1 produced detectable levels of GA_4_, whereas the SiDT1^N280Y^ mutant under the same conditions showed a stark reduction in GA_4_ production (Figure 4D). These results demonstrate that the N280Y mutation severely impairs or abolishes catalytic activity, likely through destabilizing the protein.

### 2.4 Disruption of SiDT1 Is Associated with Altered Strigolactone Signaling

**FIGURE 5:**
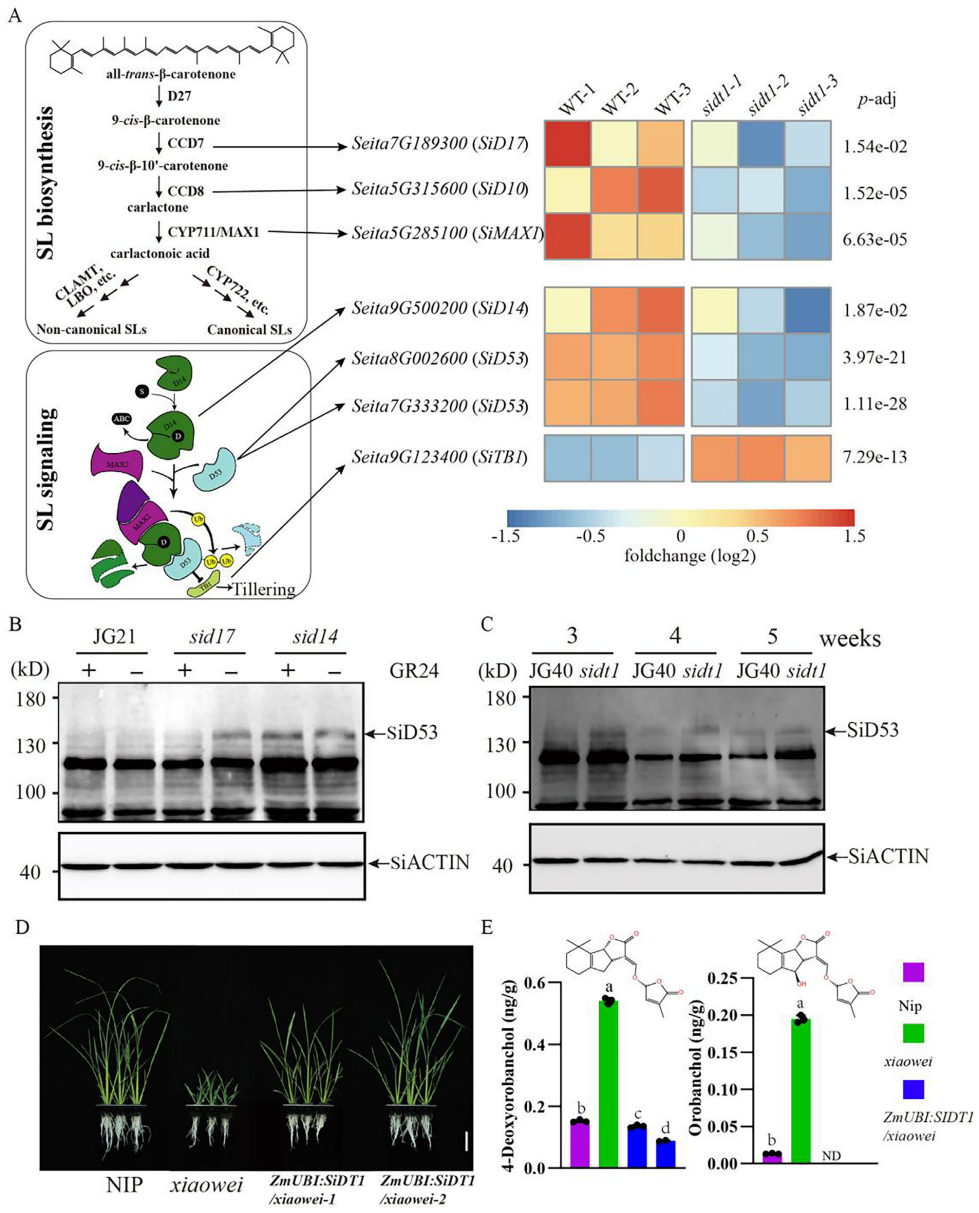
Alteration of the strigolactone pathway in *sidt1*. (A) Attenuation of the strigolactone pathway in the *sidt1* Mutant. Diagram of the SL pathway with key enzymes (left) and a heatmap of DEGs between *sidt1* and Jingu40 (right). (B) Western blot analysis of SiD53 stability in response to GR24. Wild‐type (JG21), *sid17* and *sid14* mutants treated with (+) or without (−) 10 µM GR24 for 30 min. SiD53 protein levels were detected using an anti‐SiD53 specific antibody. (C) Immunoblot showing SiD53 protein levels in JG40 and *sidt1* seedlings at 3, 4, and 5 weeks of age. Higher levels of SiD53 accumulate in the *sidt1* mutant across 3 developmental stages compared to the wild‐type. SiACTIN was used as a loading control in B and C. (D) Representative hydroponic growth phenotypes of 4‐week‐old seedlings of Nip, *xiaowei*, and two independent *ZmUBI:SiDT1* complementation lines. (E) Endogenous SL quantification. Levels of 4‐Deoxyorobanchol and orobanchol were measured in the roots of Nip, *xiaowei*, and two independent complementation lines. Data are presented as means ± SD (*n* = 3). Different letters indicate significant differences (ANOVA, *p* < 0.05).

In addition to its short stature, *sidt1* exhibits an increase in tillering. To investigate the molecular basis of this trait, we conducted RNA‐seq analysis on *sidt1* and the wild‐type Jingu40. Differential expression analysis (DESeq2, fold change > 2, adjusted *p*‐value < 0.01) identified 7,922 DEGs (Figure S6A). GO and KEGG enrichment analyses revealed shared pathways related to ribosome, DNA metabolic, and phenylpropanoid biosynthesis (Figure S6). Intriguingly, phytohormone pathway analysis showed significant alterations in both GA and SL pathways. In the *sidt1* mutant, transcript levels of key SL signaling components—including *CAROTENOID CLEAVAGE DIOXYGENASE 7* (*SiCCD7*), *SiCCD8*, *MORE AXILLARY BRANCHES 1* (*SiMAX1*), *DWARF14* (*SiD14*), and *DWARF53* (*SiD53*)—were downregulated (Figure 5A). Given that D53 functions as a repressor of SL signaling, we next examined its protein. A specific antibody against SiD53 was generated and validated for specificity (Figure S7A,B). Immunoblot analysis showed that SiD53 accumulates in both the SL biosynthesis mutant *sid17* and the SL receptor mutant *sid14* (Figure S7C–J). Notably, exogenous application of the synthetic SL analog GR24 triggered rapid degradation of SiD53 in *sid17*, confirming its canonical SL‐dependent turnover (Figure 5B). Consistent with RNA‐seq indicated perturbation of SL signaling in *sidt1*, SiD53 protein levels were elevated in *sidt1* relative to the wild type, with the difference being more pronounced during the early stage of tiller initiation (Figure 5C). We attempted to quantify endogenous SL levels in foxtail millet. However, none of the known SL species commonly detected in rice and sorghum were detectable in either the Jingu40 or the *sidt1* mutant under our experimental conditions (data not shown). This likely reflects structural divergence of SL molecules in foxtail millet rather than an absence of SL biosynthesis. In parallel, SL profiling in the rice mutant *xiaowei/d18* revealed the presence of two major SLs, orobanchol and 4‐deoxyorobanchol. Both compounds accumulated to significantly higher levels in *xiaowei/d18* compared with the wild‐type Nipponbare, showing ≈14.5‐fold and ∼3.56‐fold increases, respectively. Expression of *SiDT1* in *xiaowei* largely restored SL levels toward those of the wild type (Figure 5D,E). Collectively, these results indicate that SL signaling is impaired in *sidt1*.We also found that the downregulation of multiple GA biosynthesis genes in *sidt1*, including *ENT‐KAURENE OXIDASE* (*KO*), *ENT‐KAURENOIC ACID OXIDASE* (*KAO*), *GIBBERELLIN 20‐OXIDASE* (*GA20ox*), and *GIBBERELLIN 2‐OXIDASE* (*GA2ox*) (Figure S8). Correspondingly, the levels of GA precursors were reduced, as seen in the diminished conversions from GA12 to GA24 and from GA53 to GA19 (Figure 3).

### 2.5 *SiDT1* Maintained Lodging Resistance without Yield or Grain‐Quality Penalties at High Density

**FIGURE 6:**
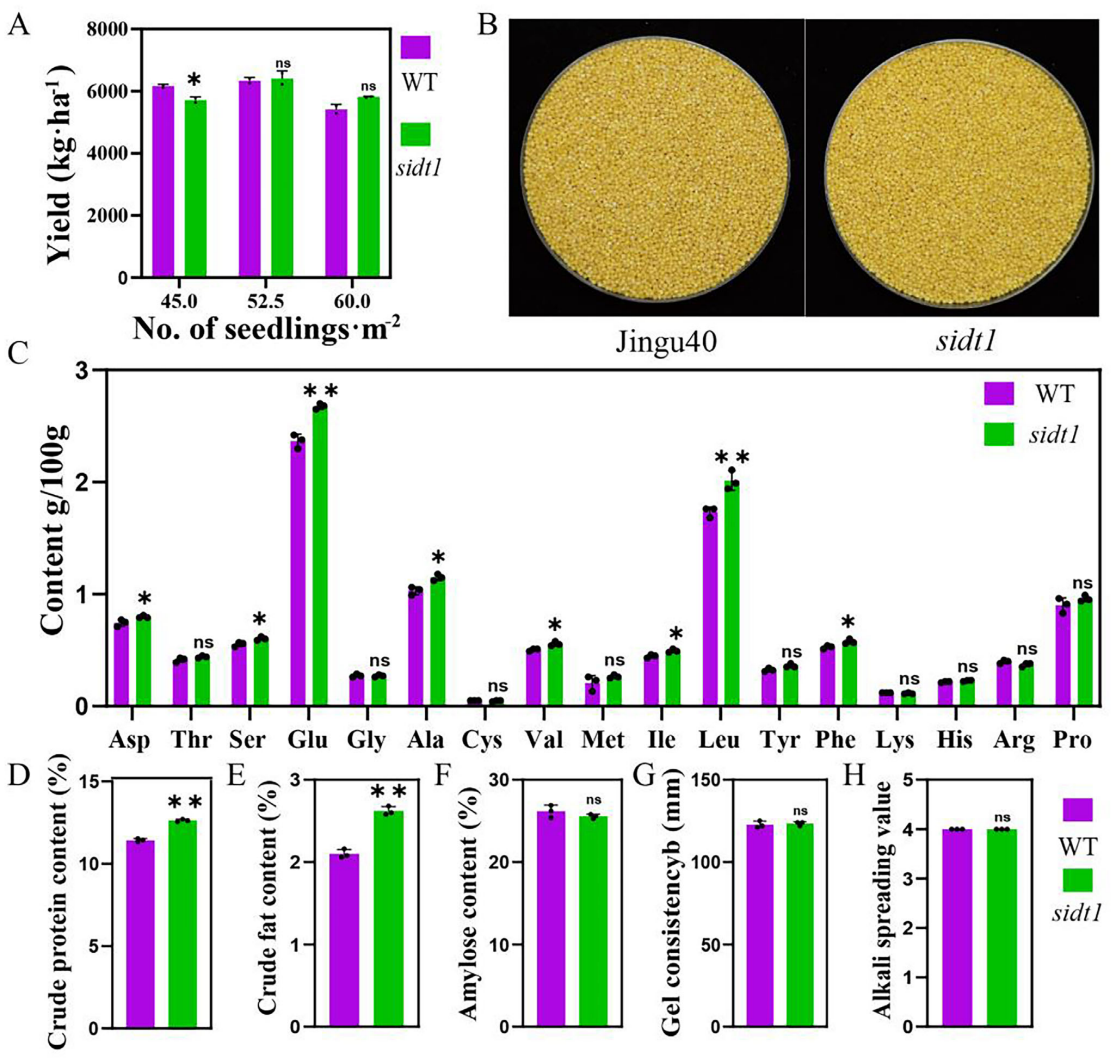
*sidt1* maintained lodging resistance without yield penalty and grain quality at high density. (A) Grain performance of Jingu40 and the *sidt1* mutant under different planting densities. Statistical analyses were performed using plot‐level biological replicates (*n* = 4, from two blocks). (B) Comparison of grain appearance quality between Jingu40 and *sidt1*. (C) Comparison of amino acid composition between Jingu40 and *sidt1*. (D–H) Comparison of crude protein (D), crude fat (E), amylose content (F), gel consisitency (G) and alkali spreading (H) between Jingu40 and *sidt1*. *, *p* < 0.05; **, *p* < 0.01.

The *sidt1* mutant exhibits a classic trade‐off: *sidt1* has smaller panicles that may lower individual‐plant yield, but its shorter height and greater tillering enable high‐density cultivation that can compensate at the plot level. To evaluate the relationship between planting density and yield potential, field trials were conducted with Jingu40 and *sidt1* at three densities (45, 52.5, and 60 plants∙m^−2^). Yield measurements were obtained from normally growing, non‐lodged plots. *sidt1* exhibited a density‐response curve with peak grain yield at 52.5 plants∙m^−2^, and its peak yield parallel that of the tall cultivar Jingu40 (Figure 6A). Both Jingu40 and *sidt1* showed a progressive yield decline as density increased, likely reflecting greater resource competition and susceptibility to lodging under dense planting. High planting density can weaken stems and intensify competition for assimilates and nutrients, which may reduce grain filling and degrade grain appearance or quality. To test this, we compared key quality traits between *sidt1* and Jingu40. No grain appearance differences were observed between Jingu40 and *sidit1* (Figure 6B). The amino‐acid profile, amylose content, gel consistency, and alkali‐spreading value (an indicator of gelatinization temperature) did not differ between the genotypes (Figure 6C–H). Together, these results indicate that *sidt1* maintains lodging resistance at high density without a measurable penalty to plot‐level yield or to grain quality, supporting its suitability for dense, mechanized cultivation. These results indicated that *sidt1* maintained lodging resistance without yield penalty and grain quality at high density.

## 3 Discussions

### 3.1 Accelerated Green Revolution Application Needed for Foxtail Millet in Modern Agriculture

The Green Revolution, conventionally dated to the 1960s, established a paradigm for enhancing crop yields through the widespread adoption of semi‐dwarf plant varieties [28]. Seminal events in this transformation included the development and application of semi‐dwarf wheat lines in Mexico in the 1940s to the 1960s, followed by the release of the semi‐dwarf rice cultivar IR8 in 1966 [26, 27, 41]. These breakthroughs drove the rapid yield gains in these staple cereals. In foxtail millet, notable progress has likewise been achieved, including the development of dwarfing germplasm such as the Ai88 lineage and the release of lodging‐resistant cultivars [34]. However, in contrast to major crops, the genetic determinants and molecular mechanisms underlying plant architecture in foxtail millet remain incompletely defined, and their systematic deployment in breeding is still limited.

Historically, its cultivation was characterized by smallholder subsistence agriculture, confined largely to marginal lands and impoverished soils where major cereals like wheat and rice could not thrive. This traditional system was defined by low external inputs and a heavy reliance on manual labor [42]. Today, however, this paradigm is undergoing a significant transition. Driven by growing recognition of its nutritional benefits and climate resilience, foxtail millet cultivation is expanding into fertile plains and is being integrated into modern, mechanized agricultural regimes [43]. Key traditional practices are being replaced by standardized operations, including increased planting densities, precision fertilizer application, and combined harvesting that replaces the labor‐intensive manual harvesting [44]. This strategic shift from fragmented, labor‐dependent production toward large‐scale, mechanized farming underscores the urgent need for semi‐dwarf plant architectures in foxtail millet that are optimized for mechanized cultivation, analogous to those underpinning the Green Revolution.

### 3.2 Recent Advances in Genetic and Germplasm Resources of Dwarf Foxtail Millet

Foxtail millet is now plausibly poised for a Green Revolution. Several dwarfing germplasm resources and their underlying genetic loci have now been identified. Interestingly, the existing dwarfing resources in foxtail millet already encompass genetic loci homologous to those that were pivotal in the Green Revolutions of wheat and rice [32]. A notable parallel is *Rht‐1*, the dominant DELLA protein in wheat. In foxtail millet, a similar mechanism involves a retrotransposon insertion that generates a constitutively active, N‐terminal‐truncated DELLA protein. This mutant protein functions as a growth repressor that is insensitive to gibberellin‐mediated degradation (Figure S9) [30]. Furthermore, the *SD1* homolog in foxtail millet, *SiSD1* (*Seita.5G404900*), carries a 1‐bp deletion in its third exon, resulting in a frameshift and premature termination at position 342 (Figure S9). Intriguingly, this position lies within a known mutation hotspot of *SD1* in rice, where amino acid changes at positions 340, 342, and 349 have been previously documented [45]. In addition, *D2*, a recessive dwarfing gene, possibly encodes a cytochrome P450 involved in phytohormone biosynthesis [33]. *SiDWARF4* encodes the ent‐copalyl diphosphate synthase (CPS) enzyme involved in gibberellin biosynthesis in foxtail millet, and its severe dwarfing effect renders the resource unusable for production [35]. Conversely, the semi‐dwarf line Ai88, valued by breeders for its compact plant architecture, became a backbone parent in foxtail millet breeding programs. Nevertheless, the causal gene(s) controlling its reduced height have yet to be identified [34].

### 3.3 Identification of *SiDT1* Facilitates the Development of Green Revolution like Traits in Foxtail Millet

In this study, we isolated a lodging resistant mutant *sidt1*. Our field evaluations of the *sidt1* mutant provide compelling evidence that it can effectively recapitulate the core agronomic principles of the Green Revolution in foxtail millet. The mutant's semi‐dwarf stature confers the essential trait of lodging resistance, a prerequisite for the high‐density planting and increased fertilizer application that underpin modern, high‐yielding agriculture. Crucially, the *sidt1* phenotype demonstrates a synergistic improvement in both height and tillering—a combination that is highly desirable for mechanized production, as it enhances yield potential through increased panicle number while maintaining a compact, manageable canopy. This coordinated control of plant architecture via a single genetic locus presents a fascinating contrast to the canonical Green Revolution trajectory. In rice, plant architecture is determined by a finely tuned balance between stem elongation and axillary bud outgrowth, which is orchestrated through antagonistic and coordinated hormonal crosstalk between GAs and SLs [46]. Exogenous application of 1 µM GA3 has been shown to reduce the endogenous SLs in rice roots by more than 20‐fold. Conversely, the application of Paclobutrazol (Pac), a potent GA biosynthesis inhibitor, results in a marked increase in SL production [47]. The core of the GA‐SL crosstalk resided in the physical interaction between their respective signaling components. Yeast two‐hybrid demonstrated that GA repressor SLR1 can physically interact with D14, the receptor for SLs [48, 49]. When SLR1 binds to D14, it could interfere with the formation of the SL‐induced D14‐D3‐D53 complex, thereby relieving SL mediated inhibition of tiller bud outgrowth and ultimately increasing tiller number. In this study, the *sidt1* mutation, by impairing GA biosynthesis, appears to orchestrate a cascade that simultaneously interrupt SL signaling, leading to a phenotype with both reduced height and increased tillering. This suggests that *SiDT1* sits at an important nexus in the hormonal network, enabling the concurrent optimization of these two key agronomic traits through a single genomic intervention. Therefore, the *sidt1* allele can become an efficient genetic solution. It bypasses the need for the multi‐step, synergistic selection of separate loci for height and tillering that characterized earlier green revolutions. By delivering an ideal plant type—short, sturdy, and highly tillered—in a single package, *sidt1* establishes a foundational and highly practical genetic resource for launching a new, streamlined Green Revolution in foxtail millet, specifically tailored for the demands of large‐scale, mechanized cultivation.

## 4 Materials and Method

### 4.1 Plant Material and Growth Conditions

The *sidt1* mutants were obtained from an EMS‐mutagenized population of the foxtail millet cultivar Jingu40 in 2021. Mapping and genetic analysis populations were generated by crossing *sidt1* with Yugu1 and Jingu40, respectively. Mutant individuals were identified from the F_2_ populations for subsequent map‐based cloning of the *SiDT1* gene. All plants were cultivated under natural field conditions at Yuci District, Jinzhong City (37°41′ N, 112°44′ E), and Ledong Town, Sanya City (18°45′ N, 109°10′ E). A severe storm event occurred on 24 July 2024 and caused foxtail millet lodging in the field. Lodging phenotypes were recorded after the storm. Individual plants that exhibited a bending angle greater than 60° from the vertical were scored as having failed to resist lodging.

### 4.2 Field Evaluation under Different Planting Densities

Field experiments were conducted to evaluate the effects of planting density on grain yield and agronomic performance of Jingu40 and the *sidt1* mutant. Prior to sowing, the experimental field was mechanically deep‐plowed and fertilized with a compound fertilizer containing 28% N, 12% P_2_O_5_, and 10% K_2_O at a rate of 600 kg ha^−^^1^. The field trial consisted of two independent blocks. Each block contained 12 plots (25 m^2^ per plot) comprising three planting‐density treatments of 45, 52.5, and 60 seedlings m^−^^2^, two genotypes (Jingu40 and *sidt1*), and two plot replicates per genotype within each density treatment. To minimize environmental variation and positional effects, Jingu40 and *sidt1* plots were arranged in an alternating pattern within each planting‐density treatment. At maturity, all plants within each plot were harvested and threshed separately. Grain yield was determined on a plot basis and subsequently converted to kilograms per hectare (kg ha^−^^1^). Statistical analyses were performed using plot‐level biological replicates.

### 4.3 Hormone Treatment and Measurement

Millet seeds were sterilized with 2.5% (v/v) sodium hypochlorite for 10 min, rinsed five times with sterile water, and then germinated for 30 h in sterile water at 37°C in darkness. Germinated seeds were transferred into a hydroponic culture medium (pH 5.5) and cultured at 28°C, 70% relative humidity under fluorescence white light (420–500 µM m^−2^ s^−1^) with a 16 h light/8 h dark photoperiod for 9 days, GA3 were supplemented as indicated concentration starting from the third day. The hydroponic solution was renewed every 3 days. The 22‐day‐old seedlings were harvested, and above‐ground tissues were immediately frozen in liquid nitrogen. Quantification of endogenous gibberellins (GAs) was performed as follows. Briefly, 100 mg (fresh weight) plant tissues were homogenized in liquid nitrogen and extracted with 90% methanol (MeOH) containing 2H2‐GAs as internal standards. The extracts were then centrifuged at 20 000 g for 15 min at 4°C and the supernatant was collected. The crude extracts were loaded onto the connected MCX–WAX cartridges (3cc, 60 mg) (Waters) to purification. After washing MCX–WAX cartridges with 90% MeOH, the WAX cartridge was separated and washed with 5% formic acid, then MeOH was used to elute GAs. After drying, the samples were redissolved in 40% MeOH for UPLC–MS/MS analysis. All detections were performed on a UPLC instrument (Waters) combined with a 6500 Qtrap MS system (AB SCIEX) equipped with an electrospray ionization (ESI) source. The MRM transitions for quantification of GAs were as follows: GA12 331.2 > 313.2, 2H2‐GA12 333.2 > 315.2; GA15 329.1 > 257.1, 2H2‐GA15 331.1 > 259.1; GA24345.2 > 257.2, 2H2‐GA24 347.2 > 259.2; GA9 315.1 > 271.1, 2H2‐GA9 317.2 > 273.2; GA4 331.1 > 257.1, 2H2‐GA4 333.1 > 259.1; GA34 347.2 > 259.2, 2H2‐GA34 349.2 > 261.2; GA53 347.2 > 303.2, 2H2‐GA53 349.2 >305.2; GA44 345.2 > 301.2, 2H2‐GA44 347.2 > 303.2; GA19 361.2 > 273.2, 2H2‐GA19 363.2 > 275.2; GA20 331.1 > 287.1, 2H2‐GA20 333.2 > 289.2; GA1 347.1 > 273.1, 2H2‐GA1 349.1 > 275.1; GA8 363.1 > 275.1, 2H2‐GA8 365.2 > 277.2.

Extraction and purification of SLs from root exudates was performed as described previously [50] with the following modifications. Briefly, the hydroponic culture medium was collected and D3‐4DO was added as an internal standard for quantification. The medium was loaded onto a pre‐conditioned Oasis HLB cartridge (Waters) for purification, washed with water and eluted with acetone sequentially. The eluate was concentrated to dryness under nitrogen gas flow. The dried sample was resuspended in 50% acetonitrile in water and analyzed using the UPLC‐MS/MS system consisting of a UPLC system (Waters) equipped with a BEH C18 column (2.1 × 100 mm, 1.7 m, Waters) and a QTRAP 6500 mass spectrometer (AB Sciex) equipped with an electrospray (ESI) source as described previously [51] with minor modification. SLs are detected in positive multiple reaction monitoring (MRM) mode. The selected MRM transition channels were 331.1 > 234.1 for 4DO, 347.1 > 233.1 for orobanchol, 361.1 > 97.1 for 4‐oxo‐MeCLA, and 334.1 > 234.1 for D3‐4DO.

### 4.4 Mapping of *SiDT1*

To map the *SiDT1* locus, genomic DNA was extracted from ≈900 F_2_ individuals displaying the mutant phenotype using a modified CTAB method. For fine mapping, SSR and INDEL markers were developed based on whole‐genome resequencing data from both parental lines. The molecular lesion in *sidt1* was identified by PCR amplification of the *SiDT1* genomic region from wild‐type and *sidt1* plants, followed by sequence alignment and comparison using ClustalW implemented in Lasergene version 7.0 (DNASTAR). Primer sequences are provided in Table S1.

### 4.5 Sequence Analysis and Phylogenetic Analysis

For SiDT1 protein sequence alignment and phylogenetic analysis, SiDT1 amino acid were subjected to a best‐BLAST match search in databases and were aligned with MEGA 12.0.14 using ClustalW for multiple sequence alignment of protein sequences. Phylogenetic trees were constructed using the NJ method with the JTT model. The reliability of the trees was evaluated by 1000 bootstrap replicates. Evolutionary rates among sites were assumed to follow a Gamma distribution (α = 1.00). The conserved 2OG‐FeII_OXY domains of the selected GA3OX family proteins were aligned and visualized using TBTools v2.390 to demonstrate the conservation of key residues.

### 4.6 Construction of Plasmids and Generation of Transgenic Plants

To construct the pUbi10‐3xFlag‐*SiDT1* plasmid, the full‐length coding sequences of *SiDT1* were amplified, then was cloned into the pDONR‐207 vector (Invitrogen) and introduced into the plant binary vector pEarleyGate‐Ubi10‐3F‐GW by LR reaction. To construct the pZmUbi‐3xFlag‐*SiDT1* plasmid, the full‐length coding sequences of *SiDT1* were amplified, then was cloned into the pDONR‐207 vector (Invitrogen) and introduced into the plant binary vector pEarleyGate‐ZmUbi‐3F‐GW by LR reaction. To generate the CRISPR‐Cas9 construct pHUE411‐*SiDT1*, pHUE411‐*SiD14*, and pHUE411‐*SiD17*, the designed guide RNA (gRNA) was ligated into the pHUE411 binary vector following standard molecular cloning procedures. For *Arabidopsis* transformation, pUbi10‐3xFlag‐*SiDT1* was verified by DNA sequencing analysis and were electroporated into *Agrobacterium tumefaciens* GV3101, which was used to transform *ga3ox1‐3* and *ga3ox1‐4* plants by the floral dip method. Successfully transformed T1 plants were selected on MS medium containing with Basta selection. For rice transformation, pZmUbi‐3xFlag‐*SiDT1* was verified by DNA sequencing analysis and were electroporated into *Agrobacterium tumefaciens* EHA105, which was used to transform *xiaowei* plants. For foxtail millet transformation, pHUE411‐*SiDT1* was verified by DNA sequencing analysis and were electroporated into *Agrobacterium tumefaciens* EHA105, which was used to transform Jingu21 variety.

### 4.7 Expression and Purification of SiDT1 and Enzymatic Assays

The *SiDT1* cDNAs was cloned into the *E.coli* expression vector pCold‐I (Takara Bio). Expression of 6His‐SiDT1 in BL21 Rosetta cells was induced with 0.1 mM isopropyl‐1‐thio‐d‐galactopyranoside at 16°C for 18 h. The recombinant fusion protein was purified using Ni Sepharose High Performance resin (Cytiva, 17526801) according to the manufacturer's instructions and quantified by the Bio‐Rad protein assay reagent. In vitro enzymatic assays were then performed. Briefly, purified SiDT1 protein (200 µL) was mixed with 2‐ketoglutarate (final concentration, 5 mM), ascorbate (5 mM), and FeSO_4_ (0.5 mM) to initiate the reaction. The reaction mixture was incubated with the appropriate gibberellin substrate at 30°C for 4 h. The reaction was terminated by adding two volumes of acetonitrile, followed by centrifugation to remove precipitated proteins. The resulting supernatant was subjected to LC–MS analysis.

### 4.8 Alfafold and Structure Prediction

The protein structure of SiDT1 was predicted using AlphaFold3. The molecular structure of gibberellin A_9_ (GA_9_) was downloaded from the PubChem database. Protein‐igand docking between SiDT1 and GA_9_ was then performed using the online platform HADDOCK 2.4, and the docked complex was further refined (light blue model). In addition, the experimentally resolved crystal structure of SiDT1 was retrieved from the Protein Data Bank (PDB) (green model). Structural comparison and analysis of the predicted SiDT1 model and the GA_9_‐binding active pocket were conducted using PyMOL. Protein stability changes upon mutation were predicted using the DUET web server (http://biosig.unimelb.edu.au/duet/). The three‐dimensional structure of the target protein was provided in PDB format, and point mutations were specified manually. DUET combines two independent stability predictors, mCSM and SDM, to generate a consensus estimate of the change in folding free energy (ΔΔG, kcal/mol) upon mutation. For each mutation, individual mCSM and SDM scores and the combined DUET prediction were extracted.

### 4.9 RNA Extraction and Sequencing

Total RNA was extracted from aerial organs of individual plants using RNAprep Pure Plant Kit (TIANGEN BIOTECH) according to the manufacturer's instructions. Total RNA (2.5 µg) was treated with DNase I and used for complementary DNA synthesis with Thermo Scientific Maxima First Strand cDNA Synthesis Kit. For transcriptome sequencing, total RNA was extracted from the whole rosette of three individual plants of each genotype at 22 DAG by RNAprep Pure Plant Kit (TIANGEN BIOTECH). The isolated total RNA was assessed for quality by Agilent Bioanalyzer. Quality and size distribution of libraries were again inspected (Agilent Tapestation). Paired end 2 × 150‐bp read‐based sequencing was performed on an Illumina HiSeq3000. The RNA‐seq raw reads were mapped to the Yugu1 T2T genome.

### 4.10 Identification of DEGs, and GO and KEGG Enrichment Analysis

Differential expression analyses were performed with DESeq2 version 1.36 (Love et al., 2014) in the R environment. The genes with adjusted *p*‐value less than 0.05 were regarded as DEGs. GO and KEGG pathway enrichment analysis was performed using R package clusterProfiler version 3.10. The raw RNA‐seq data reported in this article have been deposited in Gene Expression Omnibus, under accession number GSA: CRA037602 (https://ngdc.cncb.ac.cn/gsa).

### 4.11 Antibody Preparation

To generate a polyclonal antibody specific to foxtail millet SiD53 (Seita.8G002600), a DNA fragment encoding the N‐terminal amino acid residues 34–227 was amplified using specific primers. The resulting sequence was cloned into the pDONR222 vector via an integrative BP reaction (Invitrogen) and subsequently transferred into the pET‐55‐DEST destination vector (Invitrogen) through an LR excision reaction. The recombinant 6His‐SiD53^34‐227^ fusion protein was expressed in *E. coli* BL21 cells and purified using Ni‐sepharose resin (GE Healthcare). The truncated SiD53 protein was verified by SDS‐PAGE and then used as an antigen to immunize rabbits for polyclonal antibody production. The resulting anti‐SiD53 serum was further affinity‐purified before experimental use. For western blot analysis, the purified primary antibody was used at a 1:1500 to 1:2,000 dilution. The specificity of the anti‐SiD53 antibody was rigorously validated using total protein extracts from Jingu21, *sid17* and *sid14* seedlings.

## Author Contributions

**L.J**., **Z.K**., and **Q.Q**. conceived and designed the study. **J.L**., **J.C**., **Q.L**., **Y.Y**., **J.L**., and **Z.L**. carried out the experiments, including mutant characterization, genetic mapping, molecular analyses, and field evaluations. **Z.H**. generated the SL mutants and SID53 antibodies, and performed the western blotting. **C.P**. conducted hydroponic cultivation for rice and foxtail millet, while **J.Y**., **J.C**., and **P.X**. performed SL extraction followed by LC‐MS/MS analysis. **L.J**., **Z.K**., and **Q.Q**. analyzed and interpreted the data. **L.J**. and **J.L**. wrote the manuscript, with input from all authors. All authors discussed the results, contributed to data interpretation, and approved the final version of the manuscript.

## Conflicts of Interest

The authors declare no conflicts of interest.

## Supporting information




**Supporting File 1**: advs76506‐sup‐0001‐SuppMat.docx.

**Supporting File 2**: advs76506‐sup‐0002‐FigureS1‐S9.pdf.

**Supporting File 3**: advs76506‐sup‐0003‐TableS1‐S2.zip.

## Data Availability

The data that supports the findings of this study are available in the supplementary material of this article.
