# Supplementary material for: SiDT1 Defines Plant Architecture Reminiscent of Green Revolution in Foxtail Millet
Source: Adv Sci (Weinh). 2026 Jul 14;13(55):e76506. doi: 10.1002/advs.76506 (PMC13366370; doi:10.1002/advs.76506)
Supplement: Supplementary file 2 [file ADVS-13-e76506-s003.docx]

**Supplementary information**

***SiDT1* Defines Plant Architecture Reminiscent of Green Revolution in Foxtail Millet**

Jianzhen Lv ^1, 2 *^, Jinjin Cheng ^1 *^, Zhen Hu ^1 *^, Qian Lan ^1^, Yanjun Yang ^1^, Chengcheng Pei ^1^, Junhao Luo ^1^, Zhuang Li ^1^, Peiyong Xin ^4^, Jijun Yan ^4^, Jinfang Chu ^4^, Qian Qian ^3, #^, Zhaosheng Kong ^1, #^ and Liang Jiang ^1, #^

1 Hou-Ji Laboratory in Shanxi province, Shanxi Agricultural University, Taiyuan 030031, China

2 College of Agriculture, Shanxi Agricultural University, Taiyuan 030031, China

3 Yazhouwan National Laboratory, Sanya 572024, China

4 National Center for Plant Gene Research (Beijing), Institute of Genetics and Developmental Biology, CAS, Beijing 100101, China

^*^ These authors contributed equally.

^#^ Corresponding authors.

**Supplementary Figure 1. Analysis of agronomic traits in the *sidt1* mutant and WT under field conditions**

(A) Field performance of the sidt1 mutant and other tall varieties. Drone-captured image showing experimental plots, with the red outline highlighting the sidt1 planting area.

(B) Severe lodging of foxtail millet plants as a result of heavy rainfall. Image captured in Dongyang County, 24 July 2024.

(C) Comparison of root architecture between WT plants and sidt1 mutants. Representative images of root systems reveal noticeable differences in root structure, with sidt1 mutants displaying reduced size and branching compared to WT plants.

(D) Lodging index analysis of sidt1 mutants and WT plants. Quantitative assessment of lodging susceptibility under the same field conditions shows that sidt1 mutants exhibit a significantly lower lodging index compared to WT plants (*p < 0.05). Lower lodging index values in sidt1 mutants correspond to increased resistance to lodging. (n=8).

**Supplementary Figure 2**. **Microscopic comparison of stem tissue structure between WT and the *sidt1* mutant.**

(A) Scanning electron microscopy (SEM) images showing longitudinal and transverse views of stem tissues from WT (left column) and the *sidt1* mutant (right column) at mature stage. Alterations in cell organization and tissue compactness are evident in the mutant compared with WT.

(B) Fluorescence microscopy images of stem cross-sections from WT (left column) and the *sidt1* mutant (right column) at jointing stage. Differences in cell size and shape between genotypes were observed. Scale bars = 50 μm.

**Supplementary Figure 3. The genetic analysis of the mutant *sidt1*.**

Phenotypic segregation of tall and dwarf plants in F_2_ populations of hybrid combinations (*sidt1* x Jingu40 and *sidt1* x Yugu1*) is analyzed. The observed segregation ratios were tested using the chi-square (χ²) test at a significance level of α = 0.05, with 1 degree of freedom. The χ² values for both combinations are below the critical value of 3.84, indicating no significant deviation from the expected segregation ratio. The p-values are in the range of 0.1–0.05, supporting the goodness of fit.

**Supplementary Figure 4. Molecular identification of *SiDT1***

(A) Map-based cloning of *SiDT1*. Numbers under the markers indicate recombinants. Asterisk indicated mutation sites in *SiDT1* in the coding region and its amino acid changes. (B) Multiple sequence alignment of SiDT1 and its homologs from foxtail millet, green millet, broomcorn millet, rice, sorghum, maize, wheat, and *Arabidopsis*. The alignment was generated using ClustalW in BioEdit. Asterisks denote mutation sites within conserved amino acid residues. (C) Phylogenetic tree of SiDT1 family proteins in foxtail millet, green millet, broomcorn millet, rice, sorghum, maize, wheat, and *Arabidopsis*. The phylogenetic tree was constructed using the neighbor-joining method, with branch lengths indicating evolutionary distances.

**Supplementary Figure 5. Spatiotemporal expression profile of *SiDT1* in foxtail millet.**

Schematic representation of *SiDT1* expression patterns across developmental stages and tissues in foxtail millet. Expression levels are indicated by a color gradient (green to red), representing low to high transcript abundance, respectively. *SiDT1* is preferentially expressed in seed germination, stem, young panickle, upper leaves, and root.

**Supplementary Figure 6. Transcriptomic analysis results of the mutant *sidt1* and WT.**

(A) Volcano plot for both upregulated and downregulated differentially expressed genes (DEGs) from comparison of sidt1 versus Jingu40. The log2 FC indicates the mean expression level for each gene. Each dot represents one gene. (B) Top 10 pathway of KEGG enrichment analysis of DEGs of sidt1 versus Jingu40. The circle size of each group scaled from the metabolite number enriched in each group, the dot colors indicate log2 fold change. (C) Correlation heatmap showing the Pearson correlation coefficients among replicates of sidt1 and WT samples.

(D) Gene ontology (GO) enrichment analysis of differentially expressed genes (DEGs). The dot plot highlights significantly enriched biological processes, cellular components, and molecular functions, with the size representing the number of DEGs and the color indicating the level of significance (adjusted p-value). (E) Principal Component Analysis (PCA) revealing the distribution of mutant and WT samples in the transcriptomic data. The first two principal components explain 75.44% and 17.84% of the total variance, respectively. (F) Heatmap of DEGs between sidt1 and WT samples. Hierarchical clustering of the expression patterns demonstrates clear differences in gene expression levels, with red and blue representing upregulated and downregulated genes, respectively.

**Supplementary Figure 7. Preparation of SiD53 antibody and phenotypic characterization of strigolactone-related mutants**

(A) Schematic representation of the SiD53 (Seita.8G002600) protein structure. The diagram highlights the Clp repeat (R) domain and the AAA domain. The N-terminal peptide sequence (amino acids 34-227), used as the antigen for antibody production, is shown below. (B) Purification and verification of the recombinant 6His-siD53^34–227^ protein. SDS-PAGE analysis shows the elution fractions (lanes 1-4) of the His-tagged truncated protein. Bovine serum albumin (BSA) was used as a concentration standardto estimate the yield of the purified antigen. (C) Genomic structures and CRISPR/Cas9-induced mutations in *SiD17* and (D) *SiD14*. Black boxes and lines represent exons and introns, respectively. Targeted sequences are aligned with the wild-type (Jingu21) sequence; red letters and dashes indicate insertions and deletions, respectively. The PAM sequences are highlighted in green. (E-G) Phenotypic characterization of *sid17* mutants. (E) Representative images of Jingu21 and two independent *sid17* mutant lines. Quantification of (F) plant height and (G) tillering number. (H-J) Phenotypic characterization of sid14 mutants. (H) Gross morphology of Jingu21 and sid14 mutant lines. Quantification of (I) plant height and (J) tillering number. Data in (F, G, I, and J) are presented as means ± SD (n = 10). Asterisks indicate significant differences compared to Jingu21 (Student’s t-test; P < 0.01).

**Supplementary Figure 8. Gibberellin (GA) biosynthesis and related gene expression in *sidt1* mutant and WT.**

The GA biosynthesis pathway is depicted (left) with the involvement of plastid, endoplasmic reticulum, and cytosol. Steps in the pathway are catalyzed by key enzymes, such as CPS, KS, KO, KAO, GA20ox, GA3ox, GA2ox, and SIDT1. The molecular structures of intermediates and final GA products are illustrated. Bars represent GA content profiling for WT (purple) and sidt1 (green) samples, showing significant differences in GA levels at multiple steps of the pathway. On the right, heatmaps display the expression levels (log2 fold changes) of the genes encoding pathway-related enzymes across WT and *sidt1* samples, demonstrating differential expression patterns.

**Figure 9. Dwarfing Genes in Foxtail Millet**

Schematic overview of dwarfing genes in foxtail millet and their implications for field lodging resistance. The gene *SiDT1* has been well-characterized and identified as an effective dwarfing source suitable for agricultural production due to its ability to confer desirable plant height and lodging resistance. In contrast, other dwarfing genes, such as *SiSD1*, *D1/SiDELLA*, and *D4/SiCPS*, remain either insufficiently characterized or unsuitable for practical use due to genetic complexities. The gene Ai88 is uncharacterized and requires further investigation.

**Supplementary table 1 Primers used in this study**

**Supplementary table 2 Differentially expressed genes and corresponding *p*-values identified from RNA-seq data**
